# *Wolbachia* association with the tsetse fly, *Glossina fuscipes fuscipes*, reveals high levels of genetic diversity and complex evolutionary dynamics

**DOI:** 10.1186/1471-2148-13-31

**Published:** 2013-02-05

**Authors:** Rebecca E Symula, Uzma Alam, Corey Brelsfoard, Yineng Wu, Richard Echodu, Loyce M Okedi, Serap Aksoy, Adalgisa Caccone

**Affiliations:** 1 Department of Ecology and Evolutionary Biology, Yale University, 21 Sachem St, New Haven, CT, USA; 2 Department of Biology, University of Mississippi, University, MS, USA; 3 Yale University School of Public Health, Department of Epidemiology and Public Health, New Haven, Connecticut, USA; 4 Department of Entomology, University of Kentucky, Lexington, Kentucky, USA; 5 Faculty of Science, Gulu University, Gulu, Uganda; 6 National Livestock Resources Research Institute, Tororo, Uganda

**Keywords:** *Wolbachia*, Population structure, Sequence diversity, *gro*EL, MLST

## Abstract

**Background:**

*Wolbachia pipientis*, a diverse group of α-proteobacteria, can alter arthropod host reproduction and confer a reproductive advantage to *Wolbachia*-infected females (cytoplasmic incompatibility (CI)). This advantage can alter host population genetics because *Wolbachia*-infected females produce more offspring with their own mitochondrial DNA (mtDNA) haplotypes than uninfected females. Thus, these host haplotypes become common or fixed (selective sweep). Although simulations suggest that for a CI-mediated sweep to occur, there must be a transient phase with repeated initial infections of multiple individual hosts by different *Wolbachia* strains, this has not been observed empirically. *Wolbachia* has been found in the tsetse fly, *Glossina fuscipes fuscipes,* but it is not limited to a single host haplotype, suggesting that CI did not impact its population structure. However, host population genetic differentiation could have been generated if multiple *Wolbachia* strains interacted in some populations. Here, we investigated *Wolbachia* genetic variation in *G. f. fuscipes* populations of known host genetic composition in Uganda. We tested for the presence of multiple *Wolbachia* strains using Multi-Locus Sequence Typing (MLST) and for an association between geographic region and host mtDNA haplotype using *Wolbachia* DNA sequence from a variable locus, *gro*EL (heat shock protein 60).

**Results:**

MLST demonstrated that some *G. f. fuscipes* carry *Wolbachia* strains from two lineages. G*ro*EL revealed high levels of sequence diversity within and between individuals (Haplotype diversity = 0.945). We found *Wolbachia* associated with 26 host mtDNA haplotypes, an unprecedented result*.* We observed a geographical association of one *Wolbachia* lineage with southern host mtDNA haplotypes, but it was non-significant (p = 0.16). Though most *Wolbachia*-infected host haplotypes were those found in the contact region between host mtDNA groups, this association was non-significant (p = 0.17).

**Conclusions:**

High *Wolbachia* sequence diversity and the association of *Wolbachia* with multiple host haplotypes suggest that different *Wolbachia* strains infected *G. f. fuscipes* multiple times independently. We suggest that these observations reflect a transient phase in *Wolbachia* evolution that is influenced by the long gestation and low reproductive output of tsetse. Although *G. f. fuscipes* is superinfected with *Wolbachia*, our data does not support that bidirectional CI has influenced host genetic diversity in Uganda.

## Background

*Wolbachia pipientis* is a diverse group of α-proteobacteria found to infect many terrestrial arthropods and filarial nematodes [1], with new hosts being discovered constantly [2,3]. *Wolbachia* is currently divided into eight monophyletic “supergroup” lineages (A-H) [4], based on Multi-Locus Sequence Typing (MLST) [5].

Although this bacterium may positively influence host physiology [6,7], *Wolbachia* is best known for parasitism that alters host reproductive success, including cytoplasmic incompatibility (CI)) [8]. CI is the most studied reproductive modification induced by *Wolbachia* and results in embryonic lethality when uninfected females are crossed with *Wolbachia*-infected males. In a population composed of infected and uninfected individuals, only infected females can mate successfully with infected and uninfected males [8]. When two *Wolbachia* strains exist in a population, bidirectional CI can result in incompatibility between individuals carrying different strains, whereas individual females infected with multiple strains (superinfected) can mate with all males and produce infected progeny [9]. In both CI types, *Wolbachia* is expected to sweep through populations due to higher reproductive fitness because of the higher proportion of successful matings between infected or superinfected females relative to the uninfected ones. However, not all *Wolbachia* strains cause CI and strength of CI expression (penetrance) can be altered by *Wolbachia* density or transmission efficiency (maternal transmission fidelity) [10].

Given the influence of *Wolbachia* on host fitness, the potential impact of *Wolbachia* on host population genetic variability and geographical patterns is substantial. Since *Wolbachia* is maternally transmitted, other maternally transmitted organelles (e.g., mitochondria) hitchhike with *Wolbachia* infections [11,12]. Even though simulations indicate that CI-based spread of *Wolbachia* sweeps are more likely to involve repeated initial infections via horizontal transmission [13,14], most studies of CI-associated *Wolbachia* sweeps find it associated with low mitochondrial DNA (mtDNA) variation and with many hosts infected [15,16]. Theoretical models suggest that host dispersal or migration, and genetic background [17,18] can influence these sweeps [9,19-21]. Factors that control *Wolbachia* density, such as nutrient availability or temperature [22,23], indirectly influence CI-based sweeps, because at high *Wolbachia* densities maternal transmission fidelity and CI expression are stronger than those at low *Wolbachia* densities.

The evolutionary dynamics of *Wolbachia* spread are important for strategies aimed at vector control. In tsetse (*Glossina* spp.), the sole vectors of trypanosomes that cause Human African Trypanosomiasis (HAT), *Wolbachia* infections are common [24-26] and can cause CI [27]. In Uganda, the primary HAT vector is *G. fuscipes fuscipes*. Nuclear microsatellites and mtDNA data in this species identified discrete genetic populations and unexpected genetic breaks with complex patterns of gene flow in relatively continuous landscapes [28,29]. In Ugandan *G. f. fuscipes*, *Wolbachia* prevalence varies in most sites (6.7%-100%) with low within-individual *Wolbachia* density [26]. Contrary to expectations, *Wolbachia* prevalence was correlated to host groups defined by nuclear microsatellite data rather than mtDNA, suggesting that its spread may depend largely on host gene flow and dispersal, and not a selective sweep [26]. A possible explanation for the observed discrepancy between *Wolbachia* and host mtDNA is that there were multiple undetected *Wolbachia* strains present in each host individual, which would go undetected when only testing for infection presence.

To understand *Wolbachia* evolutionary dynamics and their relationship with host mtDNA, we used *G. f. fuscipes* from Ugandan populations of known genetic background and *Wolbachia* infection status [28-30]. We tested for the presence of multiple *Wolbachia* infections (superinfections) in single flies using four MLST loci. To test the relationship between *Wolbachia* infections and genetic variation within and among host individuals and to examine its evolutionary origin, we used sequence data from a known variable locus, *gro*EL (heat shock protein 60) [31]. We tested the hypothesis that *Wolbachia-*induced CI has shaped geographic patterns of host genetic variation and discuss how the unique life history traits of tsetse may generate unique patterns of genetic diversity in *Wolbachia*.

## Methods

### Sampling, locus selection and laboratory methods

**Figure 1:**
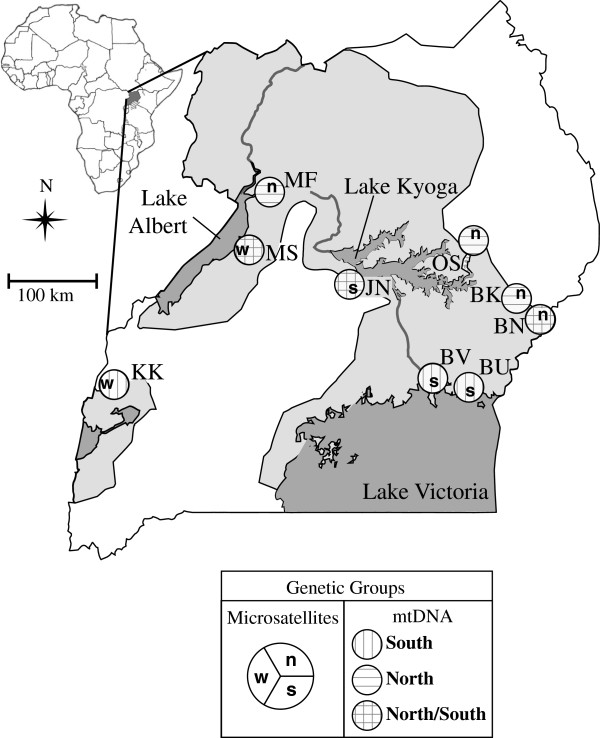
**Geographic distribution and collection sites for *G. f. fuscipes *.** Distribution, collection site and genetic group assignment based on host mitochondrial (mtDNA) and nuclear (microsatellites) data. Light gray shading illustrates the approximate geographic distribution of tsetse in Uganda. Dark gray shading indicates major water bodies. Sampling localities are illustrated as site abbreviations (See Table 1 for collection details). Circles next to sites show assignments to host mtDNA and microsatellite genetic groups as indicated by symbols in the map key. Inset map on the top left corner shows Uganda location within Africa.

Samples were selected from *Wolbachia-*positive flies with known host mtDNA and microsatellite genotypes (Figure 1, Table 1, See Additional file 1: Table S1) [26]. Details on the host genetic make-up can be found in Additional file 1. DNA was extracted from whole bodies or from ovarian tissue using Qiagen DNEasy extraction kits (Qiagen, Inc.). For the MLST examination of superinfection, four individuals (BK08033, BV10, JN6, JN8) were used from three sampling localities (BK, BV, JN, Figure 1). Between two and eight *Wolbachia*-infected *G. f. fuscipes* individuals from each of 10 Ugandan sites were selected for *gro*EL for a total of 47 flies. All individuals except BK08033 were sequenced for both MLST and *gro*EL. Due to low-density *Wolbachia* infections [26], two consecutive PCRs were performed for all target genes (See Additional file 1 for details). PCR products were cleaned and prepared for cloning using the QiaQuick Gel extraction protocol (Qiagen, Inc). For MLST, eight clones were sequenced for each individual and locus. For *gro*EL, between two and 20 clones were used for sequencing for each individual fly.

**Table 1:** **Summary of *G. f. fuscipes *collection sites, number of individuals used for *Wolbachia *sequencing, infection prevalence, and sequence diversity**

**Collection Site**	**Site Code**	**Latitude/ Longitude**	**No. of Host Individuals**	**Infection Prevalence (%)**^**1**^	**Average No. of Nucleotide Differences**	**No. of Haplotypes**	**H**_**d**_
Pallisa	BK	1.02/33.88	2	53.6 (N = 69)	4.5	6	1.000
Bunghazi	BN	0.93/33.98	5	6.7 (N = 15)	4.9	10	0.970
Busime	BU	0.25/33.97	3	29.4 (N = 34)	23.4	5	1.000
Buvuma	BV	0.31/33.30	5	60 (N = 35)	19.1	8	0.927
Junda	JN	1.33/32.74	8	65.1 (N = 43)	11.2	32	0.995
Kakoga	KK	0.37/30.28	3	35.7 (N = 28)	4.8	8	0.972
Masindi	MS	1.63/31.69	6	6.3 (N = 16)	6.0	14	1.000
Murchison Falls	MF	2.28/31.56	3	90 (N=)	7.4	19	0.983
Osuguro	OS	1.53/33.50	2	20 (N = 20)	5.3	3	1.000
Lukaya River	DRC	−4.48/15.31	5	N/A	N/A	N/A	N/A
*Gfq* Colony^2^	GF	N/A	5	N/A	N/A	N/A	N/A

^1^Infection prevalence data from [26].

^2^Collection site origin is unknown for the colony maintained at the Slovakia Academy of Science, Bratislava, Slovakia. Tissues were kindly provided by Peter Takac.

^3^Only the complete dataset was used to calculate diversity statistics in this table because the conservative dataset eliminated individuals.

Measures of *Wolbachia gro*EL nucleotide differences and genetic diversity were calculated using the complete dataset^3^. H_d_ refers to *gro*EL haplotype diversity within a site.

### Artifact and recombinant removal for *gro*EL dataset

Artificial inflation of variation in DNA sequences can arise as PCR artifact in sequence data due to infidelity and error introduced by Taq DNA polymerase. To minimize the effects of PCR artifact in the *gro*EL dataset (no variation was detected among MLST clones), each consecutive PCR amplification served as a “reconditioning PCR” [32], using a small aliquot from an initial PCR. For three individuals from a single geographic locality (JN), we amplified the same template from two independent PCR to verify strain haplotypes [32]. After sequencing, potential PCR artifacts were identified and removed using a statistical approach [33] that incorporated the GoTaq Error rate (1–7 × 10^–4^ per base pair per cycle (Promega)). We then retained all “common” sequences that were found in multiple copies within or among individuals, as their co-occurrence suggest that they are natural, rather than artificial polymorphisms (See Additional file 1 for details).

Given the high sequence diversity in the *gro*EL dataset, we used GENECONV [34], a statistical software package that detects potential recombinants by measuring whether a large proportion of DNA sequence in a pairwise comparison is more similar than expected by chance. Since we were not directly interested in recombinants, we removed any sequence from the dataset that could be a recombinant (See Additional file 1 for details). Within an individual, some recombinants may be naturally occurring rather than an artificial product arising from laboratory methods (e.g., PCR and cloning). However, there is no way to discriminate between natural and artificial recombinants, so we chose a conservative approach and excluded them, as this removal does not impact the testing of our hypotheses. As a result, our diversity measures may be underestimates, since some recombination is likely to occur between strains within a single host fly.

Excluding all *gro*EL sequences that did not occur more than once eliminated entire individual flies from our dataset and lowered the power to detect patterns of geographic variation. Complete removal of singleton sequences should not imply that *Wolbachia* is absent from that individual, but that its *gro*EL sequence is not precisely known. As such we used two *gro*EL datasets, a “complete” dataset (all sequences that could not be considered artifacts by the above methods, even if they appeared only once in the dataset), and a “conservative” dataset (only sequences found two or more times in one or more individuals). We used the complete dataset to infer the *gro*EL phylogeny, identify *gro*EL haplogroups (groups of related haplotypes), and examine the possibility of bidirectional CI. We used the conservative dataset to examine host population genetic expectations for bidirectional CI, build a haplotype network and estimate within-individual *Wolbachia* genetic diversity.

### Phylogenetic analysis and sequence diversity

For each individual fly, all clones (N = 8) from each MLST gene were identical (See Additional file 1: Table S2). Thus, we used one sequence per individual and a concatenated dataset of the four MLST genes (1635 bp) to identify *Wolbachia* superinfections. *Glossina fuscipes fuscipes Wolbachia* samples were aligned to those from [25] and from the *Wolbachia* MLST database [35] using MEGA 5.0 [36]. Phylogenetic analysis was performed using Bayesian inference and maximum likelihood. For *gro*EL, *Wolbachia* sequences from *G. f. fuscipes* were aligned to ones from other insects [37] (See Additional file 1: Table S3) in MacClade 4.08 [38,39]. jModeltest [39,40] was used to select the model of DNA sequence evolution. Phylogenetic trees were generated by maximum likelihood, as implemented in Garli 0.96b8 [41]. Branch support was generated using 1000 bootstrap replicates. Tree topologies were examined to confirm superinfections in *G. f. fuscipes*. For details of the phylogenetic analysis, see Additional file 1.

Diversity statistics were calculated on the complete and the conservative *gro*EL datasets using DNAsp [42]. To understand evolutionary relationships within *G. f. fuscipes Wolbachia* lineages at different hierarchical levels (individual flies, collection sites, and the entire dataset), we constructed haplotype networks using parsimony (TCS v1.21 [43]). Based on genetic distances within and between the networks generated from the complete dataset, we defined two *gro*EL haplogroups, “Group 1” and “Group 2” that correspond to the lineages identified in the phylogeny and divided Group 1 into subgroups (See Additional file 1: Figure S2).

### Association of *Wolbachia* haplogroups to geography and host background

We used Analysis of Molecular Variance (AMOVA [44]) on the complete and conservative datasets to evaluate the partitioning of *gro*EL genetic diversity within and among host mtDNA haplogroups and to examine whether bidirectional CI could have generated the divergent host haplogroups. Specifically, we used AMOVA to test if the diversity in *gro*EL was differentially distributed with respect to host mtDNA haplogroups. Under bidirectional CI *Wolbachia* sequences within mtDNA haplogroups should be more closely related than between haplogroups, and variance among host haplotype groups should be large and significant. In addition, we tested whether there was an association of *Wolbachia* Group 2 with southern host mtDNA haplogroups using randomization tests. Specifically, we randomly assigned the presence of *Wolbachia* Group 2 to the 47 individual flies used in our dataset to generate 100 random datasets. Then, we compared the observed number of individuals with *Wolbachia* Group 2 and the southern host mtDNA haplogroup to that in the randomized dataset.

To understand geographic relationships between host haplotypes and *Wolbachia* infection, we compared host mtDNA haplotypes and *Wolbachia* infection status. We examined *G. f. fuscipes Wolbachia* groups relative to host haplotypes and compared these samples to individuals screened for host mtDNA haplotypes [28,29] and for *Wolbachia* prevalence [26]. We specifically tested whether there was an association between host mtDNA haplotype and *Wolbachia* infection status by examining whether *Wolbachia* infected individuals were more often associated with a host mtDNA haplotype found in the contact zone between the north and south host mtDNA haplogroups. For this test, we randomly assigned *Wolbachia* infection status to the individuals screened [26] for which we had host haplotype (N = 366) to generate 100 random datasets. Then, we examined the observed proportion of flies with host haplotypes from the contact zone relative to the randomized datasets.

## Results

### Clone variation and final dataset composition

For each MLST locus from a single individual, all eight clones yielded identical sequences. In contrast, many clones yielded different sequences for the *gro*EL dataset. Following the removal of PCR and cloning artifacts, the complete dataset consisted of 47 individuals with a total of 102 *gro*EL haplotypes. The conservative dataset consisted of 37 individual flies and a total of 21 *gro*EL haplotypes. GenBank accession numbers for the 102 haplotypes are KC493415 - KC493553.

### Phylogenetic analyses for MLST and *gro*EL

**Figure 2:**
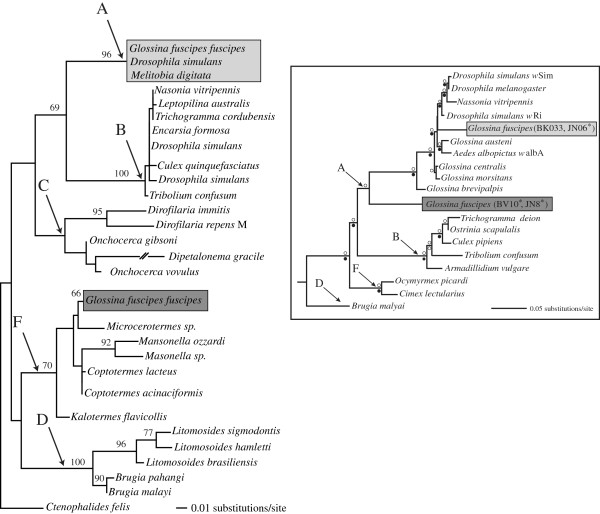
**Rooted maximum likelihood gene tree for *gro*EL and the tree inferred from four MLST loci (inset).** Arrows point to the branch that leads to each supergroup (A-F) and are labeled accordingly for both phylogenies. Shaded clades indicate the position of *G. f. fuscipes Wolbachia* sequences. Light gray shading shows the position of Group 1 sequences within supergroup A. Dark gray shading shows the position of Group 2 sequences. Individual hosts that belong to each clade are shown in the additional file. (See Additional file 1: Table S1 and Figure S1). The *gro*EL tree includes *Wolbachia* strains from *Glossina fuscipes fuscipes* and representatives from most known supergroups. For the *gro*EL tree, the outgroup was selected arbitrarily to reflect relationships in [25]. Numbers above branches are bootstrap support percentages that are above 65%. Within supergroups for *gro*EL, bootstrap support was below 65% and thus not reported. Inset (Upper right): A generalized version of the MLST phylogeny (See Additional file 1: Figure S1). An open circle above the branch indicates a Bayesian posterior probability of 1.0 and a closed circle below the branch is a bootstrap of 90% and above. The shaded boxes correspond to the shading of branches in the *gro*EL phylogeny. Individuals analyzed for both *gro*EL and MLST are indicated with an *.

Phylogenetic tree topologies inferred from the MLST and *gro*EL datasets are shown in Figure 2. The MLST dataset identified two phylogenetic *Wolbachia* lineages and suggests that one belongs to supergroup A, a group with *Wolbachia* from many insects, including other *Glossina* species [25]. The second lineage is placed outside of supergroup A, but does not align with any previously described group (Figures 2, See Additional file 1: Figure S1). Our data do not include all five *Wolbachia* MLST loci [35], and therefore do not provide a complete picture of supergroup assignment.

The *gro*EL phylogeny similarly identified two lineages, suggesting one belongs to supergroup A, but the placement of the second lineage is not supported (Figure 2). As a marker, *gro*EL evolves much more rapidly than MLST markers and may not be as informative for phylogenetic inference. We cannot directly compare the tree topology of the MLST and *gro*EL datasets to address the taxonomic placement of *Wolbachia* from *G. f. fuscipes* as our sampling differs between the datasets. However, the MLST data confirms the presence of multiple strains within *G. f. fuscipes* populations, as sequences were identical within individuals, and the *gro*EL data reveal the presence of multiple infections within individual hosts and populations.

### *gro*EL haplotype relationships and genetic diversity

**Figure 3:**
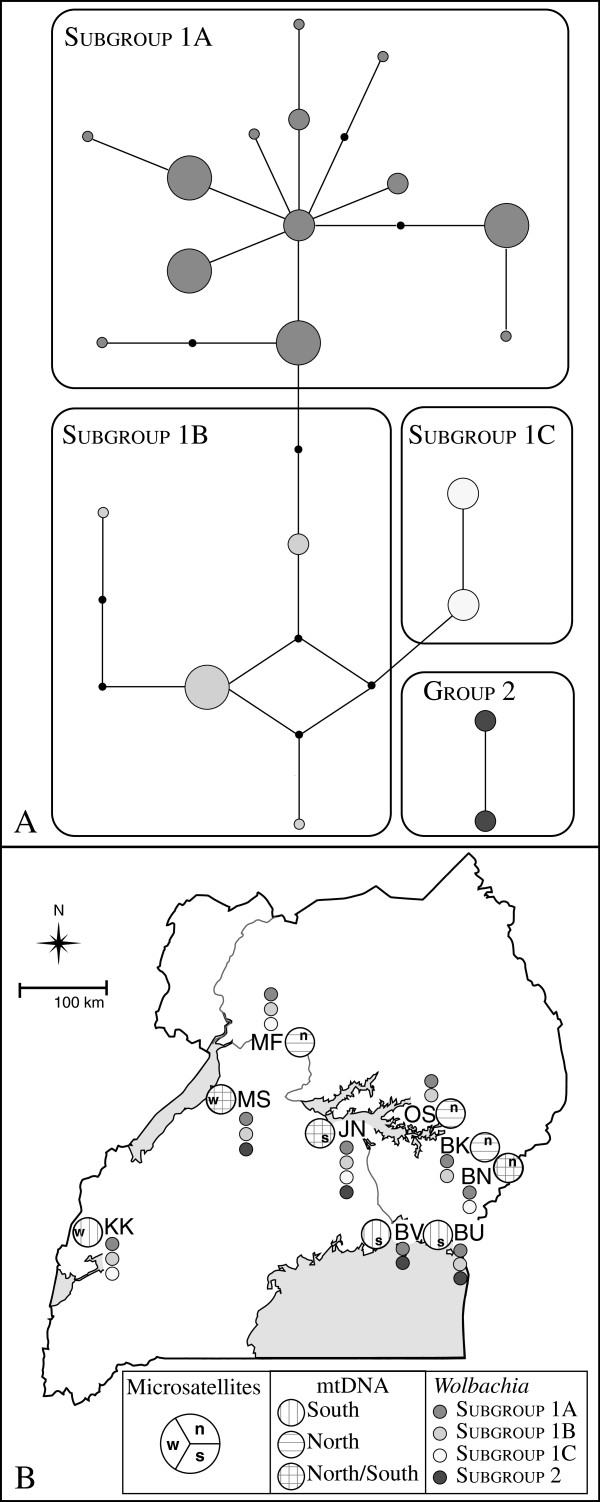
**Parsimony network and geographic locations of different haplogroups for *Wolbachia gro*EL.** Relationships among *gro*EL haplotypes, group subdivisions, and geographic distribution for the conservative dataset. **A**. TCS parsimony network for the *gro*EL haplotypes. Each circle shows one haplotype. Solid black circles (nodes) indicate number of nucleotide differences between haplotypes. Circle size represents haplotype frequency. Each branch shows one nucleotide change. Shading highlights different network subdivisions and corresponds to the haplotypes in rounded rectangles. **B**. Geographic distribution of *gro*EL haplogroups relative to previously defined genetic groups (Figure 1). Small, filled circles indicate the *Wolbachia gro*EL haplogroups represented at each site with shading corresponding to group assignment as in Figure 3A.

TCS generated two parsimony networks for both the complete and conservative datasets (Figures 3, See Additional file 1: Figure S4) corresponding to the two lineages (Group 1 and Group 2) in the phylogenetic analysis (Figure 2). Individuals within supergroup A are found in a large network (Group 1), while those outside of supergroup A are found in a smaller network (Group 2). Group 1 is subdivided into three subgroups (Subgroups 1a, 1b, 1c) for the complete dataset (For subgroup assignment details, see Additional file 1).

Measures of sequence diversity are shown in Table 2 (See Additional file 1: Table S4). In the conservative dataset, *gro*EL haplotype diversity (H_d_) is close to 1 (Table 2), ranging between 0.600 and 0.905 in the different subgroups. Nucleotide diversity (π) is highest in Subgroup 1a (π=0.0069), and approximately equal for the other groups (π=0.0018-0.00535). *Gro*EL haplotype and nucleotide diversity statistics for the complete dataset are comparable to those calculated for the conservative dataset (See Additional file 1: Table S4).

**Table 2:** **Haplotype and genetic diversity estimates for *Wolbachia gro*EL sequence groups for the conservative dataset**

**Group**	**Haplotype Diversity (H**_**d**_**)**	**Nucleotide Diversity (Π)**	**Haplotypes (N)**	**Sequences (N)**	**Segregating Sites (N)**
Entire Dataset	0.945	0.02569	21	56	52
Group “1”	0.938	0.01276	19	52	24
Subgroup “1a”	0.905	0.00691	13	36	15
Subgroup “1b”	0.644	0.00535	4	10	7
Subgroup “1c”	0.600	0.00188	2	6	1
Group “2”	0.667	0.00208	2	4	1
mtDNA N	0.957	0.01142	14	22	18
mtDNA S	0.937	0.03197	14	27	46

Groups refer to those shown in Figure 3. MtDNA N and mtDNA S refer to the host fly northern and southern mtDNA haplogroups, respectively, defined in [28,29]. *Wolbachia gro*EL sequences were assigned to the haplogroup of the individual fly from which they were obtained.

### Association of *Wolbachia gro*EL haplogroups to geography and host background

We performed an AMOVA to look for evidence of bidirectional CI in the *G. f. fuscipes* host. If bidirectional CI shaped host mtDNA diversity, we would expect to see that *Wolbachia* sequences within mtDNA haplogroups should be more closely related than between host haplogroups. The results of this analysis on the complete dataset suggest that most *Wolbachia gro*EL variation is found within (98.43%) rather than between (1.57%) tsetse host mtDNA haplogroups (p = 0.07, Table 3). The non-significant AMOVA result indicates that the host mtDNA N and mtDNA S groups are not supported by the *Wolbachia* data, a result that conflicts with the idea of bidirectional CI shaping host genetic groups. This result is also confirmed by the conservative dataset (AMOVA, p = 0.79, Table 3), as host mtDNA haplogroups have identical numbers of *Wolbachia gro*EL haplotypes (Table 2).

**Table 3:** ***Wolbachia gro*EL AMOVA results that examine the hypothesis of bidirectional CI using groups (North and South) defined by *G. f. fuscipes *mtDNA**

	**Complete dataset**				**Conservative dataset**			
	**Variance components**		**Percentage of Variation (%)**		**Variance components**		**Percentage of Variation (%)**	
Group Comparison	Among Group	Within Group	Among Group	Within Group	Among Group	Within Group	Among Group	Within Group
North *vs.* South	0.085	5.323	1.57	98.43	−0.0472	1.9411	−2.49	102.49

North and South refer to host mtDNA haplogroups.

The relationship between *gro*EL haplotypes, their group assignment, and their relationship to the host genetic variation is shown in Figure 3B. Group 1 and all three of its subdivisions (subgroups 1a-1c) occur in nearly all sites. Group 2 is mostly limited to sites with southern host mtDNA haplotypes, but it also included *gro*EL sequences from two sites (JN, MS), where both southern and northern host mtDNA haplotypes co-occur.

**Figure 4:**
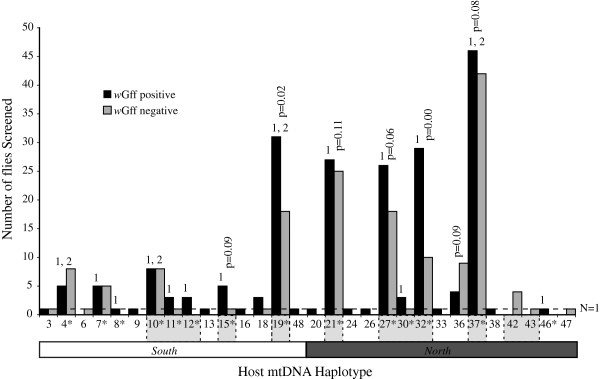
**Association between *Wolbachia *prevalence and mtDNA haplotype of the *G. f. fuscipes *host.** Association of *gro*ELfrom *Wolbachia* in *G. f. fuscipes* (*w*Gff) groups with mtDNA haplotype and tsetse infection status from Ugandan populations. The Y-axis shows the number of infected and uninfected flies (black and gray bars, respectively). The X-axis shows the host mtDNA haplotypes (numbers) and haplogropus (white and black bars for the southern and northern mtDNA haplogroups, respectively). An asterisk next to the haplotype number indicates an mtDNA haplotype associated with *w*Gff *gro*EL sequence. Gray shaded mtDNA haplotype numbers indicate those haplotypes found in, but not limited to, collection sites where both northern and southern haplotypes co-occur. A number above the bars identifies the *w*Gff group (1 and 2). P-values above haplotypes indicate whether there are more individuals infected than uninfected for a given haplotype (Binomial test).

Since *Wolbachia* is transmitted maternally, *Wolbachia* sequences should be linked with specific host mtDNA haplotypes. Most of the flies screened for *Wolbachia* had mtDNA haplotypes found in the sites with mixed host haplotypes (Figure 1, 3, 4, See Additional file 1: Table S1). However, these mtDNA haplotypes were also found in other sites. Only samples from KK, DRC and the colony, which have unique *gro*EL haplotypes, do not follow this pattern. We tested for an association between infection status and host mtDNA haplotypes in the mixed region. In 100 randomizations, we found that the proportion of infected individuals observed to have a host mtDNA haplotype found in the mixed region was not significantly different than when we randomly assigned infected status to host mtDNA haplotype (p = 0.17). *Gro*EL sequences from Group 1 are associated with all sampled host haplotypes (Figure 3B, See Additional file 1: Table S1), and thus, both host mtDNA haplogroups. In Group 2, *gro*EL sequences are only associated with four host mtDNA haplotypes: most flies had southern mtDNA haplotypes, but two flies (JN6, JN18) had the same northern mtDNA haplotype. We also tested whether the association of Group 2 with southern mtDNA host haplotypes was non-random and found a non-significant association (p = 0.16).

Figure 4 shows the association between *Wolbachia* infection status and *G. f. fuscipes* host mtDNA from a dataset from the same region [26]. The most common host mtDNA haplotypes (19, 27 and 37) have more *Wolbachia* infected individuals than other haplotypes. However, these haplotypes also occur in uninfected individuals. In *G. f. fuscipes*, many *Wolbachia*-infected individuals have host haplotypes found in the geographic region with mixed host mtDNA haplogroups, though this result is not significant (p = 0.17). In the mixed region, more individuals are *Wolbachia* positive than negative, although only three mtDNA haplotypes have significantly more infected individuals in the mixed region (black vs. grey bars*,* Figure 4). This and the association with the most common host haplotypes were consistent with the notion that infected rather than uninfected hosts should occur at higher frequency if *Wolbachia* induces CI.

## Discussion

### Intra- and inter-individual genetic diversity in *G. f. fuscipes Wolbachia* lineages

Based on *gro*EL, *G. f. fuscipes Wolbachia* lineages are diverse (Tables 1, 2), with *gro*EL haplotype diversity varying only slightly (H_d_ = 0.905-1.0) regardless of scale (e.g., dataset, lineage, group subdivision or collection site, Figure 3, 4, See Additional file 1: Table S1). When not superinfected (infected only with Group 1), *gro*EL haplotype diversity is still high (H_d_ = 0.938, Table 2). There are 102 and 21 unique *gro*EL haplotypes in the complete and in the conservative dataset, respectively. Of the 21 unique *gro*EL haplotypes, 13 were found in many individuals and sites (See Additional file 1: Table S4 and Figure S4).

Finding *Wolbachia* strain diversity in wild host populations is not unprecedented, but it has not been reported within individuals at this geographic scale. In European cherry fruit flies, *Rhagoletis*, multiple strains were found using the *wsp* gene (*Wolbachia* surface protein), but multiple strains within an individual were not reported [45]. Similarly in planthoppers, *Perkinsiella*, a number of B and F supergroup strains were identified using *wsp,* with F strains inferred to be potential recombinants [46]. While these and other studies identified high levels of *Wolbachia* diversity among individuals [47], our study found similar high levels of sequence variation at a much smaller scale, within individuals (See Additional file 1: Figure S2).

We suggest that the unique *Glossina* life history traits facilitate the identification of a transient phase of *Wolbachia* infection dynamics. Tsetse flies have a viviparous reproductive biology, where one oocyte matures and a single larva is nourished in an intrauterine environment. Females reproduce over their 3–4 month life span, producing 8–10 progeny. The low *Wolbachia* densities in *G. f. fuscipes* may reflect this viviparous biology of tsetse, since the few oocytes present in tsetse females may not necessitate retention of high *Wolbachia* densities that are required in oviparous females. However*, Wolbachia* densities in *G. f. fuscipes* were significantly lower than in the laboratory line *G. morsitans morsitans*, a species with similar life history traits. Environmental influences on *Wolbachia* densities in natural populations may be relevant and should be tested in other natural *Glossina* populations. Since *G. f. fuscipes Wolbachia* densities are very low, if a new *Wolbachia* variant arises within an individual, it is more likely to be observed and to have a proportionally larger impact on the overall genetic diversity in an individual than in a high-density *Wolbachia* infection. Thus, the peculiar tsetse life history may indirectly shape *Wolbachia* diversity within an individual host and allow the identification of variants that would otherwise not be detected.

### Origin of *G. f. fuscipes Wolbachia* infections

The observed patterns and levels of genetic diversity of the two supergroups and their co-occurrence with any one host mtDNA haplotype suggests that the origin of *G. f. fuscipes Wolbachia* (hereafter *w*Gff refers to any *Wolbachia* strain found in *G. f. fuscipes*) infections is complex and different from *Wolbachia* infection patterns reported in other studies. In insect populations that have undergone a selective sweep due to CI, *Wolbachia* infections are often associated with a single mtDNA haplotype from one or a few females [16,48]. In *G. f. fuscipes*, *w*Gff are associated with at least 26 host mtDNA haplotypes (Figure 4, See Additional file 1: Table S1) with only 15 of these host haplotypes carrying the observed *w*Gff sequence diversity. In addition, these mtDNA haplotypes are found in all *w*Gff groups. These observations could suggest that the infection in this tsetse species is ancient with unprecedented horizontal and imperfect transmission. Although this scenario is possible, it is a less likely explanation, because even with horizontal transmission we would expect to see a geographic break in the *Wolbachia gro*EL haplotypes, as we see for the host mtDNA haplotypes. An alternative hypothesis is that multiple females with different mtDNA haplotypes were initially infected. Although tests of the association between infection and mixed region host haplotypes were not significant (Randomization test, p = 0.17), all but four of these host mtDNA haplotypes are found in, but not only in, the region where northern and southern host mtDNA haplotypes co-occur (See Additional file 1: Figure S4, Table S1). This suggests that the *w*Gff Group 1 infection in Uganda may have started in the region where we observe mixed host haplotypes. Since these haplotypes are found throughout Uganda, the *w*Gff infection may have spread from there via host dispersal and subsequent gene flow. Our genetic data support this hypothesis as *w*Gff differentiation between fly populations with the two mtDNA haplogroups is low (Table 3). Furthermore, *w*Gff prevalence is associated with host genetic groups defined by microsatellite loci [26].

While our data are suggestive of a mixed region origin, the mechanism is unclear. Maternal transmission of *Wolbachia* implies independent infection of each host mtDNA haplotype. Thus our data suggest that *w*Gff in Uganda were repeatedly infected with *Wolbachia* (See Additional file 1: Table S1), a condition also supported by simulation studies as an initial transient phase in *Wolbachia* establishment in a new species [13,14], but never before observed empirically. Moreover, it is unlikely that flies dispersing from the mixed region are the sole source of *Wolbachia* infections in Uganda, as we find *w*Gff with unique host mtDNA haplotypes (KK; Figure 1) from a distinct western Uganda tsetse group defined by nuclear microsatellite data ( [29], See Additional file 1: Table S2). This suggests a second infection potentially from western Uganda. Interestingly, tsetse in Kakoga (KK) and in the Lake Victoria region (Figure 1) carrying rare mtDNA haplotypes are *Wolbachia*-infected, suggesting a relatively recent infection with a closely related *w*Gff. Since these are rare host mtDNA haplotypes, too few *w*Gff sequences are available to test this hypothesis.

The inclusion of *G. f. fuscipes* from a distant geographic location (DRC) with extremely divergent mtDNA from the Ugandan flies and from a colony population of a different subspecies (*G. f. quanzensis*) [49,50] infected by the two *Wolbachia* lineages (Figure 2) allows us to discuss three possible scenarios for the origin of the *Wobachia* infection(s). First, infection by both *Wolbachia* lineages was widespread and pre-dates the sub-species divergence. This would lead to a correlation between *Wolbachia* and host mtDNA divergence [16,51], as we see in the maternally transmitted obligate symbiont, *Wigglesworthia* in the Ugandan *G. f. fuscipes* from the same regions [52]*.* We do not see this in *w*Gff. Second, *Wolbachia* is shared between geographically disparate samples because there is extensive dispersal (and subsequent gene flow) among *G. f. fuscipes* populations. In such a case, we would expect to see *w*Gff haplotypes associated with at least one widespread host haplotype. We find *w*Gff associated with the most common host mtDNA haplotypes, but these host haplotypes are not widespread (Figure 4). Further, *w*Gff is not associated with host genetic groups defined by mtDNA variation, but with those defined by nuclear variation, whose patterns likely originated via genetic drift, not gene flow from geographically distant populations [26,28,29]. Third, it is possible that there were multiple independent infections in *G. f. quanzensis* in DRC and in *G. f. fuscipe*s in Uganda*.* Although our data appear to only support the last of these hypotheses, our sampling design does not permit us to specifically test any of these hypotheses. However, these hypotheses warrant investigation to understand *Wolbachia* infection dynamics in this species, as it can shed light on the general evolutionary dynamics of *Wolbachia* infections, which are not possible to address in other systems that do not have the viviparous life history traits of *Glossina* species.

### Relevance to CI

In the presence of CI, *Wolbachia* is expected to be associated with few high frequency host mtDNA haplotypes [9]. In combination with data from [26], we found *Wolbachia* associated with 26 host mtDNA haplotypes. Of the approximately 40 mtDNA haplotypes found in Ugandan *G. f. fuscipes*[28,29], more than half are infected with *w*Gff Group 1, and only three with *w*Gff Group 2. Indeed, the host mtDNA haplotypes infected with Group 1 are some of the most common in Uganda (Figure 4), but the low sample size for some host mtDNA haplotypes, due to low infection density, makes it difficult to draw inferences about this pattern. Interestingly, in nearly all of the high frequency mtDNA haplotypes [28,29], *w*Gff-infected individuals are more common than those that are not infected. Although these differences are not all significant, it suggests some fitness advantage for infected flies (Figure 4), consistent with occurrence of CI in *G. f. fuscipes*.

Unexpectedly, our data found *w*Gff associated with rare mtDNA haplotypes, a result also supported by *Wolbachia* prevalence data (See Additional file 1: Figure S4, [29]): in 365 flies with known host mtDNA haplotype, *w*Gff was associated with 12 extremely rare mtDNA haplotypes. CI-causing *Wolbachia* are expected to have higher fitness, driving associated host haplotypes to high frequency, as seen in some of the common *gro*EL haplotypes in our dataset (See Additional file 1: Figure S4). Contrary to the typical observation of a single female driving an infection in insect populations, theoretical studies suggest that before CI can sweep *Wolbachia* through a population, multiple independent infections must occur [13,14]. It is possible that sweeps occur in other insects too rapidly to observe these multiple, independent infections and we may have captured *Wolbachia*, even in rare *gro*EL haplotypes, due to the unique host life history.

Our genetic data do not provide evidence that bidirectional CI has shaped genetic variability in *G. f. fuscipes*[28,29]. Although Group 2 is primarily limited to southern host mtDNA haplotypes (Figure 3, See Additional file 1: Table S2), we found that this association was not significant (p = 0.16). Furthermore, two superinfected individuals have northern host mtDNA haplotypes (JN18, JN6; *gro*EL haplotype 37). This result is unexpected, if we assume solely maternal *Wolbachia* transmission, and suggests that Group 2 infections have either independently arisen in the northern host mtDNA haplotype lineage, or there is some horizontal transfer of Group 2 infections from southern *G. f. fuscipes* to individuals found in the northern mtDNA haplogroup. Horizontal transfer among different insect species must occur for *Wolbachia* to infect novel hosts, but horizontal transfer among different host species with closely related *Wolbachia* has rarely been empirically documented [53]. Since either horizontal transmission or independent infections appear to be common in *G. f. fuscipes,* genetic data may not be the ideal method to detect any form of CI as these processes may obscure host genetic patterns induced by *Wolbachia*. Furthermore, very few of our samples seem to be infected with Group 2 *Wolbachia*, potentially reducing our power to detect patterns. In contrast, we see some evidence that *w*Gff is associated with the most common haplotypes (Figure 4), suggesting a potential fitness advantage of *w*Gff. Thus, we suggest that it is crucial to examine transmission efficiency and perform laboratory mating experiments before excluding the possibility of bidirectional CI in *G. f. fuscipes*.

## Conclusions

We investigated *Wolbachia* (*w*Gff) genetic variability in the tsetse fly, *Glossina fuscipes fuscipes,* populations with known genetic composition and *Wolbachia* infection status in Uganda. Using four MLST loci, we identified two *Wolbachia* lineages, indicating superinfection of *G. f. fuscipes*. Using the variable *gro*EL gene, we confirmed the occurrence of superinfections and uncovered unprecedented sequence diversity within and between individuals. However, we do not find evidence that *Wolbachia* has influenced patterns of genetic diversity in Ugandan *G. f. fuscipes* populations through mechanisms like cytoplasmic incompatibility (CI). When compared to the host mtDNA, we found *w*Gff associated with several host mtDNA haplotypes, suggesting independent acquisition of *w*Gff infections. We hypothesize that high genetic variability in *w*Gff may be a consequence of low-density *Wolbachia* infections and the observation of multiple independent infections may be associated with the unique tsetse life history.

## Abbreviations

*w*Gff: Any *Wolbachia pipientis* strain from *Glossina fuscipes fuscipes*; *gro*EL: gene for heat shock protein 60; CI: Cytoplasmic incompatibility; MLST: Multi Locus Sequence Typing; mtDNA: mitochondrial DNA; HAT: Human African Trypanosomiasis; *G. f. fuscipes*: *Glossina fuscipes fuscipes*; *gat*B: gene for aspartyl/glutamyl-tRNA(Gln) amidotransferase, subunit B; *cox*A: gene for cytochrome c oxidase, subunit I; *fbp*A: gene for fructose-bisphosphatealdolase; *fts*Z: gene for cell division protein; AMOVA: Analysis of Molecular Variance; *G. austeni*: *Glossina austeni*; *G. brevipalpis*: *Glossina brevipalpis*; *wsp*: gene for *Wolbachia* surface protein.; H_d_: Haplotype diversity; *G. f. quanzensis*: *Glossina fuscipes quanzensis*; bp: base pairs.

## Competing interests

The authors declare that they have no competing interests.

## Authors’ contributions

RES-Wrote and revised the manuscript and performed and interpreted data analysis, UA-carried out the laboratory analyses and collected the data. CB-Collected and analyzed the MLST data, and commented on the manuscript, YW-Extracted DNA and helped collect the molecular data, RE-Provided samples, LMO-Provided samples and facilitated sample collection, SA-Conceived the project and helped write the manuscript, AC-Helped write the manuscript and provided support for the analyses. All authors read and approved the final manuscript.

## Supplementary Material

Additional file 1 **Includes the expanded methodology for laboratory methods and data analysis.** Tables of detailed sample information for *G. f. fuscipes* and other insects, primers and diversity statistics for the complete dataset. Figures show MLST phylogeny and the haplotype networks for individuals, sampling sites and the complete dataset. Click here for file
