# Supplementary material for: Wolbachia association with the tsetse fly, Glossina fuscipes fuscipes, reveals high levels of genetic diversity and complex evolutionary dynamics
Source: BMC Evol Biol. 2013 Feb 5;13:31. doi: 10.1186/1471-2148-13-31 (PMC3574847; doi:10.1186/1471-2148-13-31)
Supplement: Additional file 1 — Includes the expanded methodology for laboratory methods and data analysis. Tables of detailed sample information for G. f. fuscipes and other insects, primers and diversity statistics for the complete dataset. Figures show MLST phylogeny and the haplotype networks for individuals, sampling sites and the complete dataset. [file 1471-2148-13-31-S1.doc]

Supplemental Material

**Sampling**

Flies were selected to represent host genetic variation from northern (N), southern (S) and mixed (N/S) mtDNA haplogroups. In mixed sites, where both northern and southern host haplogroups co-occur (BN, JN, MS), between 10 and 12 individuals were sampled (Table 1). Five flies from a single site in Democratic Republic of Congo (DRC) and five from a tsetse colony of *G. f. quanzensis* (Bratislava; [1]) were also screened for comparison to the Ugandan flies. To identify superinfections using the MLST loci, four individuals (BK08033, BV10, JN6, JN8) were sequenced from three sampling localities (BK, BV, JN).

**PCR Amplification**

For *gro*EL, all PCR were performed using 1 μL of each DNA extraction, GoTaq® DNA Polymerase and 5X GoTaq Buffer, 0.2 μM primer and 3mM MgCl2. *Gro*EL sequences were amplified using WgroRev1 (5’AGA TCT TCC ATC TTG ATT CC) and either WgroFor1 (5' GGT GAG CAG TTG CAA GAA GC) (BV samples only; [2]) or a new primer designed for this study, GffWgro(F) (5' TTT GAT CGC GGT TAT C). These two primer pairs are expected to amplify a 795bp and 410 bp PCR fragments, respectively.

Due to low density of *Wolbachia* in *G. f. fuscipes*, for all *gro*EL samples two consecutive PCRs were performed to generate enough product for cloning, using 2-4 μL from the first PCR reaction as the starting template for the second reaction [3, 4]. The PCR reaction used the following cycle: Initial denaturation 94 ºC for 2 min, 38 cycles of 94 ºC for 1 min., 47 ºC for 1 min., and 72 ºC for 1 min. and a final extension at 72 ºC for 10 min.

Artificial variation in DNA sequences can arise as PCR artifact and DNA sequencing due to infidelity and error introduced by Taq DNA polymerase. To minimize the effects of PCR error, each consecutive PCR amplification described above also served as "reconditioning PCR" [5], using a small aliquot from an initial PCR. For three individuals from a single geographic locality (JN), we amplified the same template from two independent PCR to verify strain haplotypes [5].

To prepare sequences for cloning, PCR products were gel purified using the QiaQuick Gel extraction protocol (Qiagen, Valencia, CA). Cloning was performed using the pGEM-T Easy Vector System (Promega, Madison WI). Purified fragments were ligated into the pGEM-T vector plasmid and plasmids were transformed into DHα5 competent cells. Between 1-20 colonies were picked for sequencing.

DNA sequencing was performed by the Yale DAFSH using the primer for the T7 promoter (5' TAA TAC GAC TCA CTA TAG GG). Chromatograms were verified and vector sequence was removed using Sequencher v4.2.2 (Gene Codes Corp., Ann Arbor MI). Primer sequences were removed and alignments were generated manually using MacClade 4.08 (Sunderland, MA). Following alignment, all single nucleotide polymorphisms and indels (insertions/deletions) were verified in the original chromatograms. Sequences that did not have an open reading frame were discarded.

**Tests for Recombination and PCR Error assessment.**

In order to verify that the obtained *gro*EL sequences were not recombinants between multiple sequence types within an individual or PCR reaction, we used GENECONV [6], a statistical software package that detects gene conversion (recombination), the process by which a DNA segment is copied onto another DNA segment. GENECONV measures whether a large proportion of a sequence in a pairwise comparison is more similar than expected by chance. The program uses all possible pairwise comparisons in a DNA sequence alignment to identify similar regions that imply gene conversion (two sequences more similar than expected by chance), and assesses significance of similarity using permutation tests. For this study, since we were not interested in the recombinants themselves, but in the removal of potential artificial recombinants generated by laboratory methods (e.g., PCR and cloning), we took a conservative approach and removed any sequence from the dataset that potentially could be a recombinant. In order to detect the maximum number of possible recombinant sequences, GENECONV was run on the entire *gro*EL dataset, on each of the two separate *gro*EL clades identified by phylogenetic analysis (see below), and on the different *gro*EL sequences identified within each individual tsetse. Significance values were assessed using 10,000 permutations for each pairwise comparison of sequences. Sequences were removed from the dataset if the global p-value (corrected for multiple comparisons) was significant in any of the datasets described above. Since significance in any pairwise comparison indicates that particular regions of two sequences are more similar than expected by chance, any sequence that had many significant pairwise comparisons to other individuals in the dataset were also removed. We removed any sequence with 22 or more significant comparisons, an arbitrary cutoff apparent in the data. Since the datasets were large, some sequences with fewer significant pairwise comparisons were also considered recombinants if, after manual inspection of gene fragments in the alignment, sections of the alignment appeared extraordinarily similar while the remainder was substantially different. As a result of our conservative approach, diversity measures may be underestimates, since some recombination is likely to occur between strains within a single host fly. Thus, within an individual, some recombinants are actually naturally occurring rather than an artificial product arising from laboratory methods (e.g., PCR and cloning). We removed all recombinants because it is impossible to identify the source of recombination. Sequences that were removed as recombinants are shown in Figures S2 and S3.

To detect potential PCR artifacts, we used a statistical approach that accounts for the number of base differences relative to PCR product size to calculate the probability that a divergent sequence is likely to be a PCR error [4]. Using the GoTaq Error rate of 1–7 x 10^–4^ per base pair per cycle (Promega), we estimated that for PCR fragment of 410 bp (as for *gro*EL), one should expect, on average, a single substitution to arise as an artifact. However, not all groups of sequences that differed by one substitution could be eliminated as artifacts. We retained all "common" sequences that were found in multiple copies within or among individuals, as their co-occurrence suggests that they are natural rather than artificial polymorphisms. We eliminated any sequence that had one singleton mutation relative to "common" sequences, since these were more likely to be due to PCR error. Figures S2 and S3 show all sequences removed as PCR artifacts. When a host individual did not have *gro*EL sequences that appeared more than once in the dataset, we retained only sequences that were more than one singleton mutation away from a common haplotype, to avoid deleting all sequences recovered from a single individual. We refer to this data set as the “complete dataset”. A second dataset was examined without all sequences that did not appear in multiple clones or across individuals, and called it the “conservative dataset”.

**Phylogenetic Analyses**

For the MLST phylogenetic analyses, we used the best model of sequence evolution, GTR +I+γ, selected by jModeltest [7, 8]. Bayesian analyses were performed using MrBayes 3.1 [9]. Analyses were initiated from random starting trees, and two separate runs, composed of four chains, were run for 10,000,000 generations. The cold chain was sampled every 1000 generations. The burn-in period was determined by plotting the log likelihood values, and discarding trees prior to likelihood value stabilization. As a result, the first 25,000 generations were discarded. Posterior probabilities were computed for the remaining trees generated from the Bayesian analyses. ML trees were constructed using MEGA 5.0 [10], with gamma distribution rates with 1000 bootstrap replications, and the method of Jukes and Cantor as the genetic distance model.

Jmodeltest selected HKY+G as the best model of sequence evolution for the *gro*EL locus for both the Akaike and Bayesian Information Criteria. For the best tree (-lnL= -2664.8215), parameters estimated by Garli were base frequencies a=0.34185 c=0.16913 g=0.18853 t=0.30049; rate matrix: A-C=1.00000 A-G=18.99503 A-T=1.00000 C-G=1.00000 C-T=18.99503 G-T=1.00000; gamma shape parameter: 7.586. Tree scores for the 10 Garli replicates were nearly identical with the best tree differing only slightly from the worst tree (difference in -lnL=0.93). The major differences were in relationships of taxa/individuals within previously defined supergroups [2, 11] and other well-supported clades. Bootstrap proportions for relationships among supergroups are generally below 65%. In most cases, bootstrap topologies that do not support the best tree (Figure 2) also do not support monophyly of the supergroups. Thus, it is likely that the low bootstrap support is a byproduct of using a small DNA fragment from a single gene with few informative characters for phylogeny estimation.

**Defining “Group 1”subgroups from *G. f. fuscipes Wolbachia***

To define groups in “Group 1”, separate networks were constructed for each of three most densely sampled collection sites (BN, JN, MS) using the "complete dataset" (Figure S2). The relationship of each haplotype was assessed relative to the haplotype with the highest frequency in each of the networks. At all three sites, the most common haplotype was separated from the next most common haplotype by four unsampled haplotypes (substitutions), defining three subgroups within the “Group 1” lineage (Figure S4). Thus, *Wolbachia* subgroups were defined as those that contained haplotypes that were fewer than four nucleotide substitutions from the most common haplotype within the three apparent groups. Haplotypes that were equidistant from more than one group were assigned to one of the groups on the basis of their position in the network that contained all of the samples. Haplotypes from sites with fewer samples (either number of sequences or individuals) were assigned to groups only on the basis of their position in the network that contained all samples, since many sites lacked high frequency haplotypes for comparison.

References

1. Dyer NA, Lawton SP, Ravel S, Choi KS, Lehane MJ, Robinson AS, Okedi LM, Hall MJ, Solano P, Donnelly MJ: **Molecular phylogenetics of tsetse flies (Diptera: Glossinidae) based on mitochondrial (COI, 16S, ND2) and nuclear ribosomal DNA sequences, with an emphasis on the palpalis group.** *Mol Phylogenet Evol* 2008, **49:**227-239.

2. Casiraghi M, Bordenstein SR, Baldo L, Lo N, Beninati T, Wernegreen JJ, Werren JH, Bandi C: **Phylogeny of Wolbachia pipientis based on gltA, groEL and ftsZ gene sequences: clustering of arthropod and nematode symbionts in the F supergroup, and evidence for further diversity in the Wolbachia tree.** *Microbiology* 2005, **151:**4015-4022.

3. Alam UH, Hyseni, Chaz; Symula, Rebecca E.; Brelsfoard, Corey; Wu, Yineng; Kruglov, Oleg; Okedi, Loyce M.; Caccone, Adalgisa; Aksoy, Serap: **Microfauna-host interactions: implications for trypanosome transmission dynamics in *Glossina fuscipes fuscipes* in Uganda.** *Appl Environ Microbiol* in review.

4. Cummings SM, McMullan M, Joyce DA, van Oosterhout C: **Solutions for PCR, cloning and sequencing errors in population genetic analysis.** *Conservation Genetics* 2010, **11:**1095-1097.

5. Thompson JR, Marcelino LA, Polz MF: **Heteroduplexes in mixed-template amplifications: formation, consequence and elimination by ‘reconditioning PCR’.** *Nucleic Acids Research* 2002, **30:**2083-2088.

6. Sawyer SA: **GENECONV: A computer package for the statistical detection of gene conversion. .** In *Book GENECONV: A computer package for the statistical detection of gene conversion.* (Editor ed.^eds.), 1.81a edition. City; 1999.

7. Posada D: **jModelTest: phylogenetic model averaging.** *Mol Biol Evol* 2008, **25:**1253-1256.

8. Guindon S, Gascuel O: **A simple, fast, and accurate algorithm to estimate large phylogenies by maximum likelihood.** *Syst Biol* 2003, **52:**696-704.

9. Ronquist F, Huelsenbeck JP: **MrBayes 3: Bayesian phylogenetic inference under mixed models.** *Bioinformatics* 2003, **19:**1572-1574.

10. Tamura K, Peterson D, Peterson N, Stecher G, Nei M, Kumar S: **MEGA5: Molecular Evolutionary Genetics Analysis Using Maximum Likelihood, Evolutionary Distance, and Maximum Parsimony Methods.** *Molecular Biology and Evolution* 2011, **28:**2731-2739.

11. Baldo L, Dunning Hotopp JC, Jolley KA, Bordenstein SR, Biber SA, Choudhury RR, Hayashi C, Maiden MC, Tettelin H, Werren JH: **Multilocus sequence typing system for the endosymbiont Wolbachia pipientis.** *Appl Environ Microbiol* 2006, **72:**7098-7110.

12. Abila PP, Slotman MA, Parmakelis A, Dion KB, Robinson AS, Muwanika VB, Enyaru JC, Okedi LM, Aksoy S, Caccone A: **High levels of genetic differentiation between Ugandan Glossina fuscipes fuscipes populations separated by Lake Kyoga.** *PLoS Negl Trop Dis* 2008, **2:**e242.

13. Beadell JS, Hyseni C, Abila PP, Azabo R, Enyaru JC, Ouma JO, Mohammed YO, Okedi LM, Aksoy S, Caccone A: **Phylogeography and population structure of Glossina fuscipes fuscipes in Uganda: implications for control of tsetse.** *PLoS Negl Trop Dis* 2010, **4:**e636.

Table S1-Detailed summary of individuals screened for *Wolbachia* genetic variation using *gro*EL. “Collection site” refers to Table 1 and Figure 1. “Individual” refers to the tsetse individual. “Tissue source” indicates if a whole fly (W) or the reproductive tract (R) were used to extract DNA. “MtDNA haplogroup” and "mtDNA haplotype” follow [12, 13]. “*Wolbachia* clones” shows the number of sequenced *gro*EL clones. “Clones retained” shows the clones kept following tests for PCR error and recombination and comprise the "complete dataset". “Unique haplotypes” refers to the adjusted number of clones with unique haplotypes. “*Wolbachia* Group/Subgroup” reports the *gro*EL haplotype assignment from Figures 3 and S4. Asterisks indicate flies with greater than 10 sequences collected.

| Collection Site | Individual | Tissue Source | mtDNA haplogroup | mtDNA haplotype | *Wolbachia* clones (#) | Clones retained (#) | Unique haplotypes (#) | *Wolbachia* Group/Subgroup |
| --- | --- | --- | --- | --- | --- | --- | --- | --- |
| PALISA | PALISA6 | W | N | -- | 4 | 4 | 3 | 1a |
|  | PALISA7 | W | N | -- | 4 | 4 | 3 | 1a, 1b |
| BN | BN2.1 | R | N | 21 | 5 | 5 | 4 | 1a |
|  | BN6.3 | R |  | -- | 5 | 1 | 1 | 1a |
|  | BN6.4 | R | N | 27/30 | 4 | 3 | 3 | 1a |
|  | BN8.2 | R | S | 19 | 4 | 2 | 2 | 1a |
|  | BN8.4 | R | S | 19 | 7 | 4 | 2 | 1c |
| BU | BU17 | W | S | 19 | 1 | 1 | 1 | 2 |
|  | BU24 | W | S | 19 | 2 | 2 | 2 | 2 |
|  | BU4 | W | S | 15/19 | 4 | 2 | 2 | 1a, 1b |
| BV | BV10 | W | S | 19 | 5 | 4 | 3 | 1a, 2 |
|  | BV34 | W | S | -- | 5 | 3 | 3 | 1a, 2 |
|  | BV7 | W | S | 8 | 5 | 4 | 1 | 1a |
|  | BV8 | W | S | 4 | 4 | 3 | 2 | 1a, 2 |
|  | BV9 | W | S | 19 | 4 | 4 | 2 | 1a, 2 |
| DRC | DRC2 | W | N/A | 39 | 3 | 2 | 1 | 1a |
|  | DRC3 | W | N/A | 40 | 5 | 4 | 4 | 1a, 1c, 2 |
|  | DRC6 | W | N/A | 40 | 4 | 3 | 1 | 1c |
|  | DRC7 | W | N/A | 40 | 4 | 3 | 3 | 1c |
|  | DRC8 | W | N/A | 41 | 1 | 1 | 1 | 1c |
| COLONY | GF1 | W | N/A | -- | 1 | 1 | 1 | 1c |
|  | GF2 | W | N/A | -- | 5 | 4 | 3 | 1a, 1c, |
|  | GF4 | W | N/A | -- | 4 | 2 | 2 | 1c, 2 |
|  | GF5 | W | N/A | -- | 3 | 3 | 3 | 1b, 2 |
|  | GF6 | W | N/A | -- | 3 | 3 | 2 | 1c |
| JN | JN16* | R | S | 11 | 11 | 8 | 8 | 1a, 1b |
|  | JN17 | R | S | 11 | 6 | 6 | 5 | 1a, 1b |
|  | JN18 | R | N | 37 | 6 | 5 | 4 | 1a, 1b, 2 |
|  | JN2 | R | S | 11 | 5 | 4 | 4 | 1a, 1c |
|  | JN20 | R | S | 12 | 3 | 2 | 2 | 1a |
|  | JN28 | R | N | 37 | 1 | 1 | 1 | 1a |
|  | JN6* | R | N | 37 | 11 | 9 | 8 | 1a, 1b, 1c, 2 |
|  | JN8* | R | N | 37 | 11 | 10 | 2 | 1a, 1b |
| KK | KK09004 | W | S | 7 | 5 | 4 | 4 | 1a, 1b, 1c |
|  | KK09007 | W | S | 46 | 3 | 3 | 2 | 1a |
|  | KK09024 | W | S | 7 | 6 | 6 | 3 | 1a |
| MF | MF1 | R | N | 37 | 5 | 5 | 5 | 1a |
|  | MF15 | R | N | 37 | 6 | 5 | 4 | 1a, 1c |
|  | MF4 | R | N | 37 | 6 | 5 | 5 | 1a, 1b, 1c |
| MS | MS1.4 | R | S | 10 | 5 | 4 | 2 | 1b |
|  | MS1.10 | R | S | 10 | 5 | 4 | 4 | 1a, 1b |
|  | MS11.1 | R | N | 32 | 5 | 5 | 5 | 1a |
|  | MS11.2 | R | N | 32 | 5 | 3 | 3 | 1a, 1b |
|  | MS6.6 | R | N | 32 | 6 | 4 | 4 | 1a |
|  | MS6.7 | R | S | 10 | 5 | 4 | 4 | 1a, 1b, 2 |
| OS | OS09018 | W | N | 37 | 2 | 2 | 1 | 1b |
|  | OS09006 | W | N | 37 | 4 | 4 | 2 | 1a, 1b |

Table S2 Locus information and primers used to amplify *Wolbachia* loci in *G. f. fuscipes*.

| Gene | Product | Name | Primer Sequence (5’- 3’) | Gene length (bp) | Fragment size (bp) | Reference |
| --- | --- | --- | --- | --- | --- | --- |
| *gro*EL | heat shock protein 60 | WgroFor1  WgroRev1 | GGTGAGCAGTTGCAAGAAGC  AGATCTTCCATCTTGATTCC | 320 | 320 | [2] |
|  |  | GffWgro(F) | TTTGATCGCGGTTATC |  | 320 | This paper |
| *gat*B | aspartyl/glutamyl-tRNA(Gln) amidotransferase, subunit B | gatB_F1  gatB_R1 | GAKTTAAAYCGYGCAGGBGTT  TGGYAAYTCRGGYAAAGATGA | 1,425 | 369 | [11] |
| *cox*A | cytochrome c oxidase, subunit I | coxA_F1  coxA_R1 | TTGGRGCRATYAACTTTATAG  CTAAAGACTTTKACRCCAGT | 1,551 | 402 | [11] |
| *hcp*A | conserved hypothetical protein | hcpA_F1  hcpA_R1 | GAAATARCAGTTGCTGCAAA  GAAAGTYRAGCAAGYTCTG | 741 | 444 | [11] |
| *fts*Z | cell division protein | ftsZ_F1  ftsZ_R1 | ATYATGGARCATATAAARGATAG  TCRAGYAATGGATTRGATAT | 1,197 | 435 | [11] |
| *fbp*A | fructose-bisphosphatealdolase | fbpA_F1  fbpA_R1 | GCTGCTCCRCTTGGYWTGAT  CCRCCAGARAAAAYYACTATTC | 900 | 429 | [11] |

Table S3 Representative taxa from *Wolbachia* supergroups for the *gro*EL locus. Species indicates the invertebrate species from which *Wolbachia* was isolated. Accession no. refers to GenBank Accession number. Supergroup assignment refers to the group assignment in the original papers.

| Species | Accession No. | Dataset | Supergroup |
| --- | --- | --- | --- |
| *Leptopilina australis* | AY714802.1 | *gro*EL | B |
| *Tribolium confusum* | AY714798.1 | *gro*EL | B |
| *Drosophila simulans* | AY714800.1 | *gro*EL | B |
| *Culex quinquefasciatus* | AY714804.1 | *gro*EL | B |
| *Drosophila simulans* | AY714799.1 | *gro*EL | B |
| *Encarsia formosa* | AY714797.1 | *gro*EL | B |
| *Trichogramma cordubensis* | AY714803.1 | *gro*EL | B |
| *Nasonia vitripennis* | AY714796.1 | *gro*EL | B |
| *Onchocerca volvulus* | Y09416.1 | *gro*EL | C |
| *Dipetalonema gracile* | AJ609658.1 | *gro*EL | C |
| *Onchocerca gibsoni* | AJ609652.1 | *gro*EL | C |
| *Dirofilaria repens* | AJ609653.1 | *gro*EL | C |
| *Dirofilaria immitis* | AJ558023.1 | *gro*EL | C |
| *Ctenophalides felis* | AJ609659.1 | *gro*EL | -- |
| *Brugia malayi* | AF373870 | *gro*EL | D |
| *Brugia pahangi* | AJ609654.1 | *gro*EL | D |
| *Litomosoides brasiliensis* | AJ609655.1 | *gro*EL | D |
| *Kalotermes flavicollis* | AJ609660.1 | *gro*EL | D |
| *Coptotermes acinaciformis* | AJ627384.1 | *gro*EL | F |
| *Coptotermes lacteus* | AJ627385.1 | *gro*EL | F |
| *Masonella sp.* | AJ628412.1 | *gro*EL | F |
| *Mansonella ozzardi* | AJ609657.1 | *gro*EL | F |
| *Microcerotermes sp.* | AJ628411.1 | *gro*EL | F |
| *Mellitobia digitata* | AY714808.1 | *gro*EL | A |
| *Drosophila simulans* | AY714806.1 | *gro*EL | A |

Table S4 Haplotype and genetic diversity estimates for *Wolbachia* *gro*EL sequence groups and subgroups for the complete dataset. "Group" refers to those shown in Figure 3. MtDNA N and mtDNA S refer to the host fly northern and southern mtDNA haplogroups, respectively, defined in [12, 13]. *Wolbachia* *gro*EL sequences were assigned to the haplogroup of the individual fly from which they were obtained.

| Group | Haplotype Diversity (H_d_) | Nucleotide Diversity (Π) | Haplotypes (N) | Sequences (N) | Segregating Sites (N) |
| --- | --- | --- | --- | --- | --- |
| Entire Dataset | 0.9906 | 0.03620 | 102 | 138 | 128 |
| Group "1" | 0.988 | 0.01765 | 89 | 123 | 98 |
| Subgroup "1a" | 0.977 | 0.01116 | 51 | 75 | 61 |
| Subgroup "1b" | 0.954 | 0.00988 | 21 | 27 | 30 |
| Subgroup "1c" | 0.971 | 0.00976 | 17 | 21 | 24 |
| Group "2" | 0.981 | 0.01054 | 13 | 15 | 21 |
| mtDNA N | 0.994 | 0.02750 | 50 | 58 | 93 |
| mtDNA S | 0.987 | 0.03903 | 45 | 58 | 76 |

Figure Legends

Figure S1 Phylogeny of *Wolbachia* based on the concatenated dataset for the four MLST loci: *gatB*, *fbpA, ftsA,* and *coxA* (1635 bp). Parentheses on *G. f. fuscipes* samples indicate the individual used for MLST. Parentheses on other samples indicate *Wolbachia* sequence types (ST) and are available from the *Wolbachia* MLST database and the reference, if available for the sequences. Bayesian posterior probability (top) and Maximum likelihood boostrap (bottom) values are indicated at major nodes if they were supported by both clustering algorithms (i.e., Maximum likelihood and Bayesian). Letters and arrows indicate the branch that leads to each known supergroup (A-F) and are labeled accordingly. Parentheses following the *G. f. fuscipes* samples indicate sample site location.

Figure S2 Haplotype networks for each individual tsetse host in nested boxes. Individuals are clustered by sampling locality (boxed). Circles indicate haplotypes, solid black circles indicate haplotypes not sampled. Size of the circle indicates the frequency that a haplotype was sampled. Text beside the circles shows the corresponding individual and clone of a given haplotype. PCR Error and Recombinants are filled with yellow and red, respectively. Open circles indicate samples that were retained. Numbers next to circles indicate the clone number represented by the haplotype.

Figure S3 Network reconstructions for each sampling locality (enclosed in a rectangle with locality name) used for defining the supergroup A lineage subdivisions. Circles represent haplotypes and circle size shows their relative frequency. Small black circles represent haplotypes not sampled and thus, number of substitutions between haplotypes. Text beside circles shows the corresponding individual and clones of a given haplotype. Shading highlight subdivisions and colors indicate haplotypes inferred to be either PCR Error or Recombinants (see Legend).

Figure S4 TCS parsimony network showing relationships among *gro*EL haplotypes, groups and subgroups and geographic distribution for the complete dataset. Each circle represents a single unique haplotype. Solid black circles (nodes) indicate haplotypes not sampled. Size of the circle represents the frequency of the haplotype. Text beside the circles shows the corresponding individual and clone of a given haplotype. Each branch represents a single nucleotide change. Shading highlights different network subdivisions and corresponds to the haplotypes encircled by the rounded rectangles.

Symula et al. Fig. S1

Symula et al. Fig. S2

Symula et al. Fig. S3

Symula et al. Fig. S4
